# Supplementary material for: LDL-Cholesterol-Lowering Effects of a Dietary Supplement Containing Onion and Garlic Extract Used in Healthy Volunteers
Source: Nutrients. 2024 Aug 22;16(16):2811. doi: 10.3390/nu16162811 (PMC11356941; doi:10.3390/nu16162811)
Supplement: Supplementary file 1 [file nutrients-16-02811-s001.zip › nutrients-3135984-supplementary.pdf]

Table S1. Detailed descriptions of participants' tobacco use status, alcohol consumption and physical activity habits.

**Smoker:**	
	25% mild (less than 3 cigarettes a day)
	50% moderate (less than 10 cigarettes a day)
	25% heavy (10 or more cigarettes a day)
**Former smoker:**	
	95% quit more than 10 years ago
	3% quit between 1 to 10 years ago
	2% quit less than 1 year ago
**Alcohol consumption:**	
	95% drink only on weekends and special occasions
	5% drink 4 or more days a week
**Physical activity:**	
	98% exercise 2 or more days a week
	2% exercise occasionally or 1 day a week

Detailed descriptions of participants' tobacco use status, alcohol consumption and physical activity habits to give a clearer picture of the study population. We ensured that there was an equal distribution between groups for these characteristics to maintain the integrity and comparability of our study.
